# Squid-Inspired Tandem Repeat Proteins: Functional Fibers and Films

**DOI:** 10.3389/fchem.2019.00069

**Published:** 2019-02-21

**Authors:** Abdon Pena-Francesch, Melik C. Demirel

**Affiliations:** ^1^Center for Research on Advanced Fiber Technologies, Materials Research Institute, Pennsylvania State University, University Park, PA, United States; ^2^Department of Engineering Science and Mechanics, Pennsylvania State University, University Park, PA, United States

**Keywords:** tandem repeat, protein, sustainability, fibers, thin films, self-healing, soft photonics, 2D nanocomposites

## Abstract

Production of repetitive polypeptides that comprise one or more tandem copies of a single unit with distinct amorphous and ordered regions have been an interest for the last couple of decades. Their molecular structure provides a rich architecture that can micro-phase-separate to form periodic nanostructures (e.g., lamellar and cylindrical repeating phases) with enhanced physicochemical properties via directed or natural evolution that often exceed those of conventional synthetic polymers. Here, we review programmable design, structure, and properties of functional fibers and films from squid-inspired tandem repeat proteins, with applications in soft photonics and advanced textiles among others.

## Introduction

Many globular and fibrous proteins have repetitions in their sequences or structures. However, a clear relationship between these repeats and their contribution to the physical properties in materials remains elusive. Exquisite knowledge of structure-property relationships in proteins will allow the design of materials with programmable properties that have novel functionalities. The scientific progress in this field is growing rapidly as we understand the effects of long-range order (i.e., the frequency and form of repetition) on macromolecular complexity. Here, we summarize recent studies on a specific class of tandem repeat proteins inspired by squid ring teeth as a model material system by combining expertise in nanoscale materials science, molecular biology, and protein physics.

Proteins based materials are composed of large biomolecules consisting of long chains of amino acids that fold and hierarchically assemble into complex and well-defined structures (Bechtle et al., 2010; Hu et al., 2012). The amino acid sequence of proteins can be precisely tuned since a defined sequence is genetically encoded in the DNA. This allows absolute control over stereochemistry, sequence, and chain length. Proteins are heteropolymers, which have exact molecular weight and assemble into complex hierarchical structures (defined by the sequence), whereas conventional homopolymers mainly form random coil conformations, and have statistical distributions of molecular weights and sequences. The precise control of the primary amino acid sequence regulates the assembly into the hierarchical structures, and ultimately governs the resulting physical, chemical, and biological properties of the material (e.g., mechanics, stability, activity, etc.) (Mann and Jensen, 2003; Jenkins et al., 2008). Additionally, proteins are naturally biocompatible with cell-interactive properties and tailored biodegradability, which makes them a material of interest for biomedical applications.

Naturally occurring proteins can be directly extracted from the native organisms. Due to lack of abundance or programmability requirements, recombinant expression in a variety of hosts has been the choice for the production of proteins. Over the past couple of decades, researchers have explored a wide range of expression systems for the high-yield production of proteins such as bacteria (Lewis, 2006; Xia et al., 2010; Heidebrecht and Scheibel, 2013), yeast (Fahnestock and Bedzyk, 1997; Cereghino et al., 2002), plants (Scheller et al., 2001), mammalian cell lines (Lazaris et al., 2002), and transgenic organisms (Tomita et al., 2003). Genetically modified *Escherichia coli* (*E. coli*) is the most established suitable host for industrial-scale production due to commercially available of expression vectors and well-understood genetics (Schmidt, 2004; Terpe, 2006; Heidebrecht and Scheibel, 2013). In addition, recombinant expression of engineered artificial genes allows for the biosynthesis of proteins with specified combinations of the 20 natural amino acids and a variety of unnatural amino acids (>100), expanding the possibilities of protein design (Link et al., 2003; Johnson et al., 2010).

## Repetitive Structural and Fibrous Proteins

**Figure 1:**
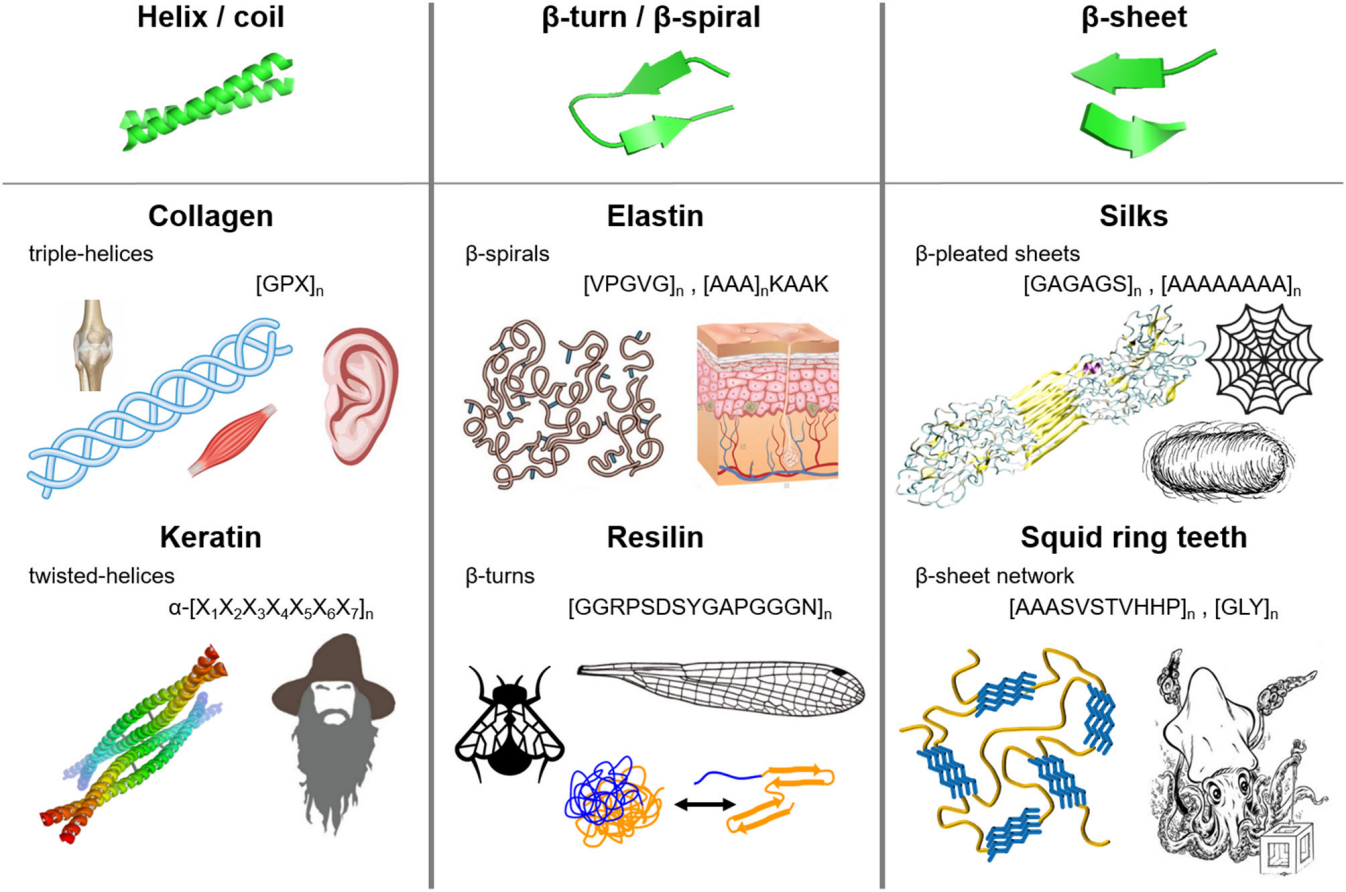
Molecular architecture and repetitive sequences of fibrous protein polymers: (i) coiled-coils (e.g., collagen and keratin), (ii) β-turns/spirals (e.g. elastin and resilin), and (iii) β-sheets (e.g., silks and squid ring teeth).

Nature has evolved many functional materials across the animal and plant kingdom with hierarchical structures across the mesoscale and nanoscale that are built from protein building blocks. Many of the protein-based biological building blocks converged into a same family of structures despite evolving separately. Figure 1 summarizes the major structural elements found in repetitive protein polymers, namely coiled-coils, β-sheets, and β-turns/spirals, which are briefly reviewed below.

### Helical Coiled-Coil Proteins

Coiled-coils are bundles of α-helices that are twisted into a superhelix, and are usually found in nature in extracellular matrix proteins (Lupas et al., 1991; Lupas, 1996; Kohn et al., 1997). α-helix structures (first predicted by Pauling et al., 1951) consist of a helical arrangement of the protein backbone, typically with 3.6 amino acid residues per turn of the helix. Each α-helix is stabilized by hydrogen bonding between the backbone amino and carbonyl groups and those in the next turn of the helix, leaving the amino acid side chains in the outer shell of the helix (Voet and Voet, 2011). Coiled-coil structures are abundant in naturally occurring proteins such as collagen and keratin.

Collagen composes up to 30% of whole human body protein content, and is found throughout fibrous tissues such as tendons, cartilage, ligaments, and skin (Di Lullo et al., 2002). Its major functions are scaffolding, tissue assembly, and repair (Kadler et al., 2007; Fratzl, 2008). Fibrillar collagens (types I, II, and III) have [GPX]_n_ repeats, where X is usually occupied by proline and/or hydroxyproline (Brodsky and Baum, 2008; Shoulders and Raines, 2009). The GPX repeat forms left-handed α-helices that intertwine into right-handed triple helices (Kadler et al., 2007; Fratzl, 2008). Recombinant hydroxyproline (Luo and Tong, 2011) collagen has been synthetized for tissue engineering (e.g., corneal substitution, cartilage replacement) as well as biosensing and therapeutics applications (Toman et al., 2000; Teles et al., 2010; San et al., 2016).

Keratin, on the other hand, forms helical filaments that can be found in epithelial and epidermal appendages such as hair, nails, horns, hooves, wool, and skin (Rouse and Van Dyke, 2010). Due to its high sulfur content (i.e., disulfide bonds cross-link the coils), keratin is highly insoluble and mechanically strong, contributing to waterproofing and strengthening of hair and epidermal tissues (Wang et al., 2016). α-keratins have a repeating hepta-peptide, α-[X_1_X_2_X_3_X_4_X_5_X_6_X_7_]_n_, sequence that form right handed α-helices dimers (Wang et al., 2016). Within the repeat unit, the first, fourth, fifth, and seventh positions are located at the hydrophobic interface between two α-helices, while the second, third, and sixth positions are exposed to the outside environment. The first and fourth amino acids of the heptapeptide are non-polar (usually occupied by leucine, hence the name “leucine zippers”) (Landschulz et al., 1988), and they form the hydrophobic plane along each helix and dominate the inter-helical hydrophobic interactions (Wang et al., 2016). The hydrophobic planes align between helices to form dimers, which are further stabilized by hydrogen bonding and crosslinking of cysteine residues via disulfide bonds (Fraser et al., 1976; Rouse and Van Dyke, 2010; Wang et al., 2016). Common heptapeptide units such as EVSALEK, KVSALKE, EIAALEK, KIAALKE, VAALEKE, and VAALKEK have been used as supramolecular cross-linkers in keratin-inspired coiled-coil protein-based materials (Wang et al., 2016). The hierarchical assembly of coiled-coil domains has been explored in the development of biomedical hydrogels. Since the aggregation of coils is driven by hydrophobic inter-helical interactions, a variety of stimuli can disrupt the association and trigger stimuli-responsive behaviors: temperature, ionic strength, pH, and denaturing buffers (Petka, 1998; Xu et al., 2005). In addition, the mechanical properties and association kinetics can be tailored by adjusting the amino acid composition of the heptapeptides (different side chains protruding from the helix) (Dooling and Tirrell, 2016; Dooling et al., 2016). Control of association and dissociation of coiled domains has led to shear thinning and self-healing protein-materials, which are used as injectable biomedical hydrogels (Ifkovits and Burdick, 2007; Wong et al., 2009; Olsen et al., 2010).

### β-Turn/β-Spiral Elastic Proteins

Most elastic proteins are intrinsically disordered but contain a high fraction of β-turns and polyproline structures (Tatham and Shewry, 2000, 2002; Shewry et al., 2003; Roberts et al., 2015). β-turns are small secondary structures involving four amino acids that form intramolecular hydrogen bonding (Muiznieks and Keeley, 2010; Voet and Voet, 2011). Elastin, which is found in the extracellular matrix and connective tissue (especially in human skin), is composed of water-soluble monomers that aggregate into non-soluble constructs. It has a common hydrophobic domain VPGVG that exhibits a lower critical solution temperature (LCST). Above this temperature, the hydrophobic domains interact and aggregate into β-turn structures separating from the soluble phase (Urry and Parker, 2003). Additionally, elastin has lysine residues that, after posttranslational modification into allysine, chemically cross-link the hydrophobic domains yielding non-soluble stretchable elastin (Pinnell and Martin, 1968; Yeo et al., 2011). The ability to control and modify specific amino acid residues along the backbone of elastin provides programmability of hydrophobicity and aggregation kinetics, yielding thermo-responsive elastic materials. Hence, elastin-like proteins (ELPs) that mostly derive from the VPGVG repeat have been used in drug delivery of pharmaceuticals, tissue engineering, biosensing, and protein purification (Simnick et al., 2007; Chow et al., 2008; Qi and Chilkoti, 2014).

Resilin is another elastic protein with high content of β-turn and β-spiral structures. In nature, it is found in the wing hinge, jumping pads, and vocal cords of some insects (Kim et al., 2007; Qin et al., 2012). Their high frequency functions require very elastic and resilient materials (e.g., up to 95% resilience) (Qin et al., 2012). Resilin proteins have three main components that function cooperatively as an energy storage/release mechanism: (i) exon I, water-lubricated elastic domain, (ii) exon II, cross-linked to chitosan frame, and (iii) exon III, energy-storing component (Qin et al., 2012). Resilin has GGRPSDSYGAPGGGN hydrophilic repeats of glycine and proline providing chain flexibility, which are stabilized via dityrosine cross-linking (Tamburro et al., 2010; Qin et al., 2012). Resilin has been expressed recombinantly, and dityrosine cross-linking has been achieved through enzymatic chemistry and photo-cross-linking (Elvin et al., 2005). Synthetic resilin-like proteins were used in tissue engineering as degradable scaffolds with cell-binding domains (Li et al., 2011).

Flagelliform silk, which is the connecting lines of a spider web absorbing the energy of impacting prey, is an elastic protein that has high content of β-turns and β-spirals (Hayashi and Lewis, 1998). Ninety percent of flagelliform silk is composed of GPGGX motifs (common β-turn motif) that can be cross-linked via disulfide bonds through incorporating cysteine residues (Heim et al., 2010).

### β-Sheet-Structured Proteins

β-sheet structures are formed by laterally-connected strands of peptides with hydrogen bonding interaction between the backbone carbonyl oxygen and the amino hydrogen atoms, and provide stability and mechanical strength through strong intermolecular interactions. Multiple β-strands are arranged into an extensive hydrogen-bonding network with their neighboring strands, forming crystal-like domains in the protein matrix. Silk is the most extensively studied β-sheet-structured fibrous protein. Spun by a variety of insects (including 45,000 different kinds of spiders), it serves as predatory and protective material, with tensile strength (i.e., ~700 MPa and ~1 GPa for *Bombyx mori* silkworm and *Araneus diadematus* spider silks respectively) and toughness (i.e., approaching 160 MJ m^−3^ depending on the silk type) surpassing those of high-end synthetic polymers such as Kevlar (Altman et al., 2003; Vendrely and Scheibel, 2007; Hardy et al., 2008). Silkworm silk fibroin consists of heavy and light chain, which are bound through disulfide bridges and glycoproteins. The heavy chain consists of GAGAGS hydrophobic motifs that associate into stiff pleated β-sheets, while the hydrophilic light chain provides flexibility (van Hest and Tirrell, 2001; Kundu et al., 2013). Spider silk is composed of several types of proteins such as dragline silk (i.e., main frame of spider webs) and flagelliform silk (i.e., elastic connecting silk, which is rich in β-turns and spirals). Dragline silk, spun by the major ampullate gland, contains polyalanine and GA repeats that form pleated β-sheets, and helical and turn domains that provide elasticity (Fu et al., 2009; Hardy and Scheibel, 2010). The hydrophobic interactions in the polyalanine domains drive the formation of β-sheets, and govern the semicrystalline morphology and mechanical properties of the material (Hayashi et al., 1999; Keten et al., 2010; Cetinkaya et al., 2011). Silkworm silk is obtained directly from silkworm cocoons, and it has been used historically in textiles and paper since 3000 B.C., and in the two last decades for biomedical applications such as wound dressings, drug delivery, tissue repair, biophotonics (Omenetto and Kaplan, 2008, 2010; Pritchard and Kaplan, 2011; Kundu et al., 2013). Spider silk based repetitive proteins are also recombinantly expressed (Spiess et al., 2010) due to the fact that spiders are difficult to farm and direct expression of the full protein is difficult due to its large size (Xia et al., 2010).

Curli proteins are β-sheet-rich proteins that are found in amyloid fibers and in *E. coli* and *Salmonella* biofilms (Knowles and Buehler, 2011; Evans and Chapman, 2014). Amyloid fibers have recently received significant research efforts in order to understand their aggregation mechanism and their potential role in neurodegenerative diseases such as Huntington's, Parkinson's, and Alzheimer's diseases (Prusiner and Hsiao, 1994; Lednev et al., 2006). The core of amyloid fibers has S(X)_5_QXGXGNXA(X)_3_Q repeating motifs that aggregate into cross-β-sheet structures (i.e., β-sheet-turn-β-sheet) (Evans and Chapman, 2014). Synthetic curli proteins were used for the development of functional biofilms with site-specific binding, abiotic, and adhesive properties (Nguyen et al., 2014; Botyanszki et al., 2015).

A recently discovered structural protein from squid ring teeth presents opportunities for developing multifunctional films and coatings for adhesives, wound dressing, electronic devices, sensing, smart repairable textiles, abrasion-resistant microfibers, and other applications. We review them in the next section in detail.

## Squid Ring Teeth Based Fibers and Films

**Figure 2:**
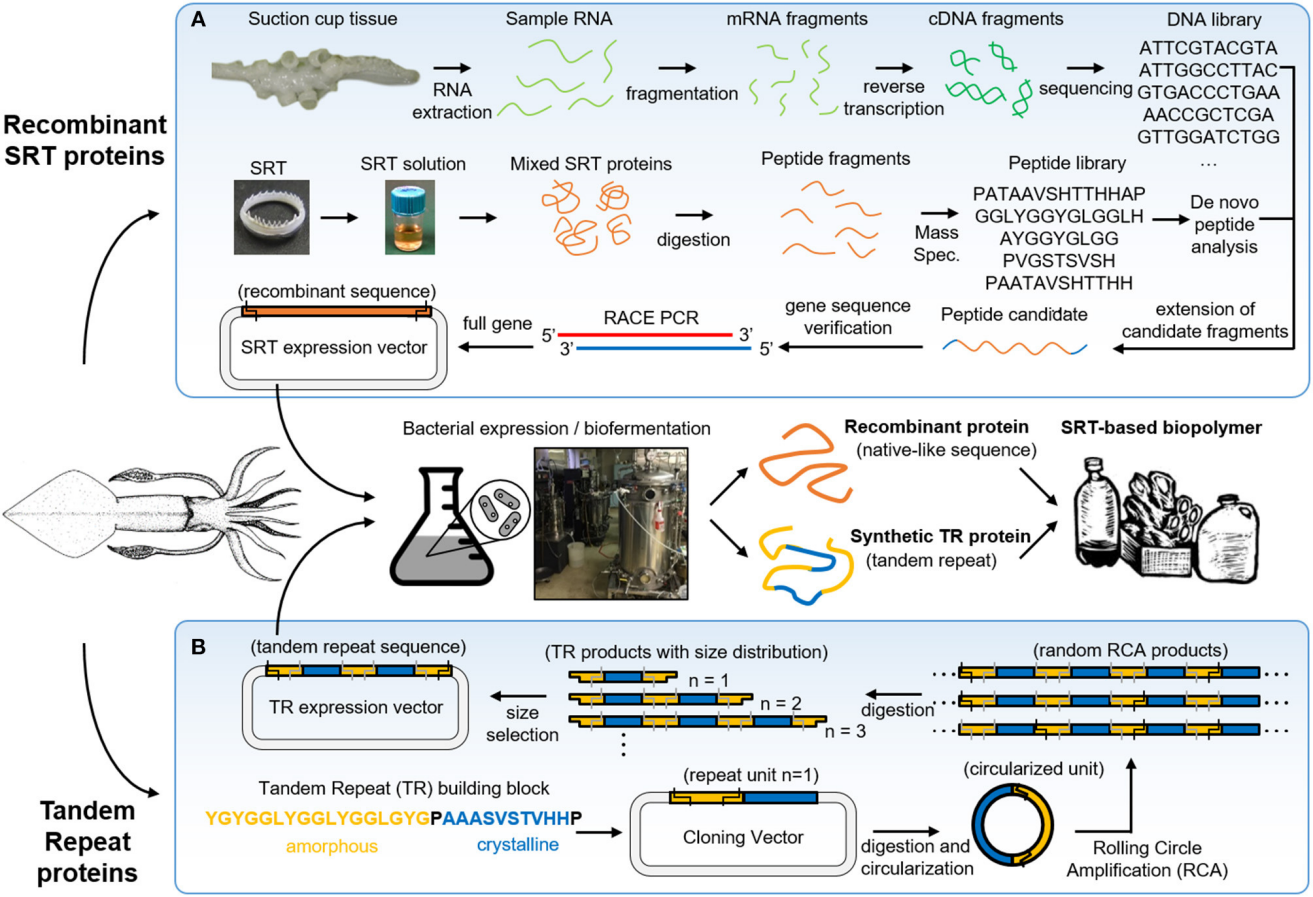
Squid ring teeth (SRT) are found inside suction cups of squid species, which are composed of a protein complex. Proteins can be extracted directly from natural sources, or expressed **(A)** recombinant and **(B)** *de novo* via biosynthetic routes (genetically modified bacteria, yeast). Adapted with permission (Pena-Francesch et al., 2018b). Copyright 2018 American Chemical Society.

SRT are predatory appendages located inside the suction cups of squid species, used to strongly grasp prey (Williams, 1910). These teeth are composed of a naturally occurring protein complex (Nixon and Dilly, 1977) with mechanical properties in the range of 4–8 GPa (Miserez et al., 2009), and have recently gained attention in the biomimetics field due to their interesting structure and properties (Pena-Francesch et al., 2018a). SRT proteins can be extracted directly from squid suction cups, or can be also biosynthetically produced using heterologous expression in bacteria after genome sequencing (Figure 2A) (Pena-Francesch et al., 2014b, 2018a).

### Design and Synthesis

Biosynthetic expression of SRT proteins presents several advantages over direct extraction from the natural source: (i) sustainability (no depletion of squid) (Pena-Francesch et al., 2018a), (ii) scaling-up and industrial scalability (Pena-Francesch et al., 2018a), (iii) programming the composition and length of the polypeptides (Pena-Francesch et al., 2018c), (iv) control over the nanostructure by manipulating the amino acid sequence (Jung et al., 2017), and (v) incorporation of functional polypeptide modules by *de novo* design of amino acid sequences (Jung et al., 2016). Direct extraction of natural SRT protein from squid tentacles is limited to availability and cost of natural sources. Global capture production in the major fisheries over the last decade is 2.2 annual million ton approximately (including all major squid species for human consumption) (Arkhipkin et al., 2015). One can make a rough estimation of the overall cost by considering a 0.5 kg average squid (for example, *Loligo vulgaris*) that can produce 100 mg of SRT (Roper et al., 1984). If SRT were extracted from all captured squids, this would yield approximately 220 ton of SRT annually. With an efficient and low cost system for extracting SRT (without contaminating the food processing so it could be later sold for human consumption), we could estimate a minimum extraction cost of $1 per squid and $10 per gram of SRT. If an efficient and low cost system for extracting SRT without damaging the rest of the animal were to be designed, the production cost could be approximated to a minimum of a $1 per squid ($0.7/squid and $0.3/squid for current collection and handling price, assuming that the whole squid could be sold for human consumption after the process without any additional cost). This would give an estimated minimum production cost of $10 per gram of SRT by means of direct extraction from the animal. Compared to the production cost of a high end polymer ($10/kg) and to the large production volume in the polymer industry (300 million tons produced per year globally) (PlasticsEurope, 2018), the volume and production costs of SRT by direct extraction are several orders of magnitude inferior and more costly. Therefore, large-scale production is necessary for economically feasible and sustainable protein-based bioplastic production for engineering and medical applications. Genetically modified *Escherichia coli* (*E. coli*) bacteria is the most established suitable host for industrial-scale protein expression due to the availability of expression vectors and well-understood genetics (Schmidt, 2004; Terpe, 2006; Heidebrecht and Scheibel, 2013). However, two major challenges remain for the production of high molecular weight repetitive proteins: the aggregation of proteins in inclusion bodies (limiting the yield), and an expensive infrastructure for scale-up (Landschulz et al., 1988). Currently, we are producing synthetic SRT protein in 80L fermenters with yields of ~1 g/L, purity of >90%, and an estimated minimum cost of ~$100/kg. We note that higher protein production yields (>10 g/L) and lower cost ~$10/kg could be achieved by optimizing the expression process (Edlund et al., 2016).

SRT proteins have a segmented amino acid sequence with alternating crystalline and amorphous regions (i.e., reminiscent of block copolymers) (Sariola et al., 2015). The amorphous regions include flexible chains rich in glycine and tyrosine amino acids, while the crystalline regions (β-sheet nanocrystals) are formed by Ala-rich segments stabilized by hydrogen bonding, separated by proline residues (Guerette et al., 2013). Tandem repetition observed in SRT proteins results in a network morphology, where β-sheets act as physical cross-linkers, and provide mechanical strength for the polymeric material (Pena-Francesch et al., 2018c). In order to fully replicate the chemistry, structure, and properties of natural SRT proteins, a new design strategy for the expression of SRT-inspired polypeptides with precise control of the sequence, segment length, and molecular weight is required. Recombinant DNA technology has been successfully used in the synthesis of tandem repeats of naturally occurring peptides (Kempe et al., 1985; Lee et al., 2002; Rao et al., 2005; Hou et al., 2007; Wang and Cai, 2007). However, current methods for DNA polymerization have major limitations: (i) they require multiple sequential steps, (ii) they cannot be run in parallel, and (iii) they do not offer precise tunable control over a range of molecular weights (Amiram et al., 2011). The synthesis of high molecular weight repetitive sequences is complicated due to genetic instability (Meyer and Chilkoti, 2002; Tang and Chilkoti, 2016), and researchers often opt for protein cross-linking from a tandem repeat monomer (which introduces defects in the protein structure such as cyclic chains) (Dimarco and Heilshorn, 2012; Li et al., 2015; Yang et al., 2017). Overcoming these limitations, rolling circle amplification (RCA) offers a one-step method to synthetize repetitive proteins from a DNA monomer with precise control over the number of repeats (Amiram et al., 2011). Recently, our team used protected digestion rolling cirle amplification (PD-RCA) to synthetize a library of squid ring teeth—tandem repeat (SRT-TR) proteins with controlled number of repeat units (Figure 2B), which is summarized here (Jung et al., 2016). A DNA sequence encoding for a SRT-inspired “monomer” was constructed based on consensus sequences derived by inspection of the native SRT proteins of several squid species: *Loligo vulgaris, Loligo pealei, Todarodes pacificus, Euprymna scolopes, Dosidicus gigas, Sepioteuthis lessoniana*, and *Sepia esculenta* (Guerette et al., 2013, 2014; Jung et al., 2016). A representative sequence consisted of a crystal-forming segment of PAAASVSTVHHP, and a disordered segment of STGTLSYGYGGLYGGLYGGLGYG was selected to create tandem repeat proteins inspired by squid ring teeth proteins. The “monomer” DNA construct was digested and circularized (i). The circularized “monomer” DNA is used as template as polymerase rolls around it, forming random RCA products (linear oligomers) (ii). The RCA products are digested, yielding a library of TR products comprised of an integer number of repeats of the TR “monomer” gene (iii). The TR products are separated by size via electrophoresis, and specific TR DNA oligomers can be selected by direct extraction from the electrophoresis matrix (iv). The selected DNA oligomers are then ligated to an expression vector to create an expression library for TR protein synthesis (v). Hence, this method can generate protein libraries comprising TR polypeptides (with the same building block sequence) with a specified number of repeat units.

### Physicochemical Properties

**Figure 3:**
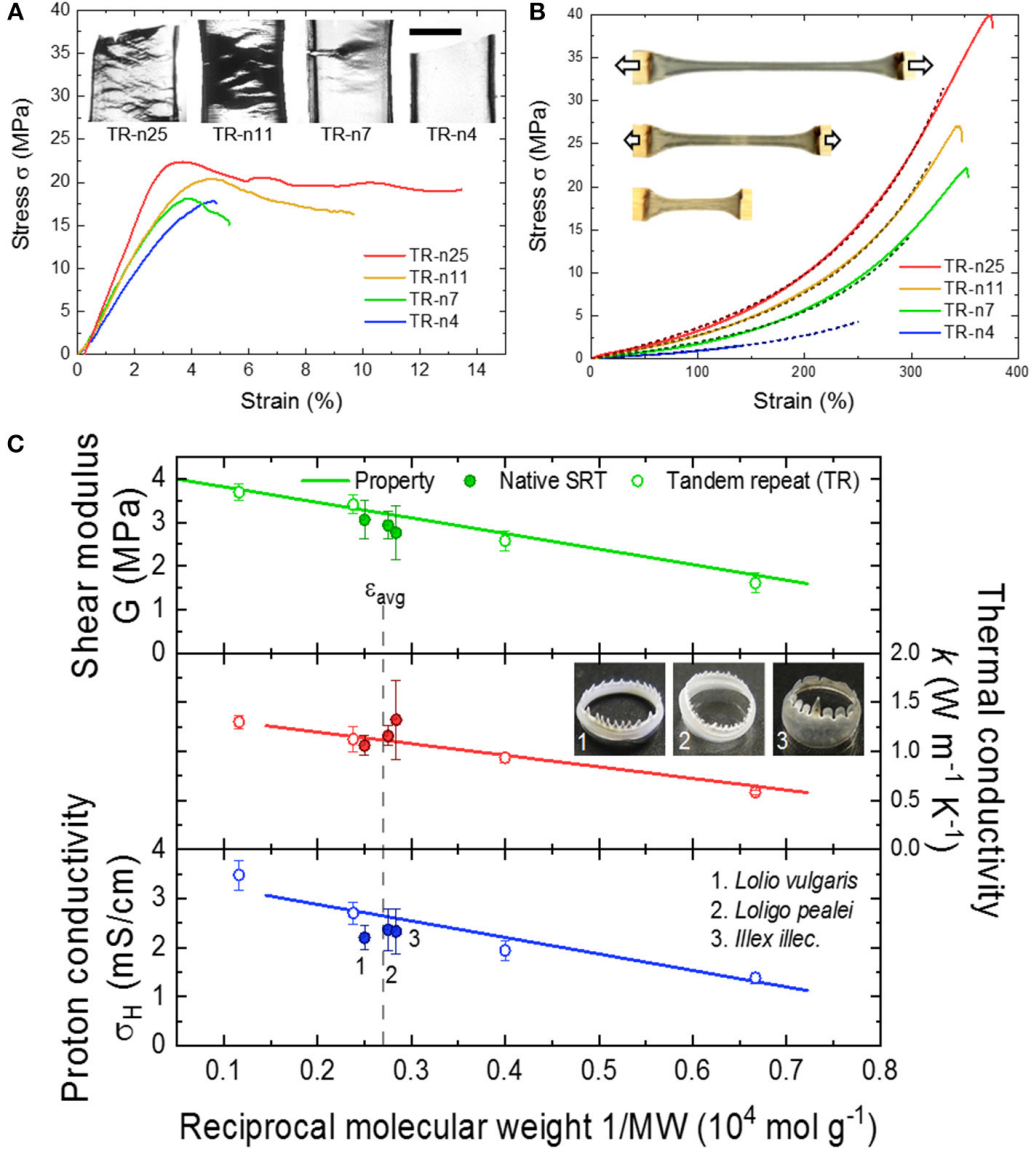
Tunable properties of tandem repeat proteins inspired by SRT. Mechanical behavior in **(A)** dry and **(B)** wet conditions is highly dependent on tandem repetition. Reproduced with permission (Jung et al., 2016; Pena-Francesch et al., 2018c). Copyright 2016 National Academy of Sciences. Copyright 2018 American Chemical Society. **(C)** Mechanical and conducting (proton and thermal transport) properties of SRT-based materials are programmable and controlled by the amino acid composition, nanostructure, and network morphology. Reproduced with permission (Pena-Francesch et al., 2018b,c; Tomko et al., 2018). Copyright 2018 American Chemical Society. Copyright 2018 Nature Publishing Group.

The physical and chemical properties of SRT proteins are governed by: (i) the amino acid composition, (ii) the secondary structure content (e.g., random coils, α-helices, β-sheets, etc.), and (iii) the overall network morphology. For example, SRT proteins contain 11% histidine amino acids (pKa 6.0) which regulate the protein charge as function of pH (i.e., positive at low pH, neutral at pH 7, and negative at high pH) (Pena-Francesch et al., 2014a). Furthermore, histidine residues contribute to proton conductivity in SRT proteins, as recently demonstrated in self-healing highly proton conductive protein films (Pena-Francesch et al., 2018b). The secondary structure of SRT film also has a strong impact on the material properties. Ordered domains like β-sheet structures provide mechanical strength (e.g., SRT and silk fibroin are β-sheet-rich structural proteins with modulus in the GPa range), while disordered domains provide elasticity and flexibility to the material (e.g., similar to disordered resilin in the wing tendon of insects) (Cheng et al., 2010; Guerette et al., 2013; Yarger et al., 2018). The secondary structure content does not only influence the mechanical properties of SRT-based materials, but also their thermal (Jung et al., 2017), conducting (Pena-Francesch et al., 2018b), and optical properties (Yilmaz et al., 2016, 2017). The morphology of disordered domains also plays an important role in defining the bulk properties of protein-based SRT materials. From this perspective, SRT is considered as a network protein gel. The disordered amorphous strands can adopt different arrangements including tie-chain conformations (i.e., connecting two neighboring β-sheet nanocrystals) or defective conformations such as dangling ends and loops (i.e., considered as topological defects). Therefore, by tuning the number of tandem repeats in SRT film, it can exhibit network morphologies ranging from a perfect network (rich in connecting tie-chains) to a defective network (rich in topological defects) (Pena-Francesch et al., 2018b,c). Effective strands contribute to stress bearing and transport throughout the bulk material, and their density should be maximized in order to improve the material properties. In SRT and other tandem repetition proteins, the effective strand density scales with reciprocal molecular weight, and consequently the mechanical and transport properties (thermal conductivity, proton conductivity) can be optimized by adjusting the molecular weight and tandem repetition (Figure 3) (Jung et al., 2016; Pena-Francesch et al., 2018b,c; Tomko et al., 2018). Therefore, SRT proteins offer programmable properties through the fine control of the amino acid sequence, nanostructure, and network morphology, which can be all encoded in the DNA sequence of SRT-inspired synthetic polypeptides (Pena-Francesch et al., 2014b, 2018a,b; Jung et al., 2016; Tomko et al., 2018).

### Fabrication and Processing

**Figure 4:**
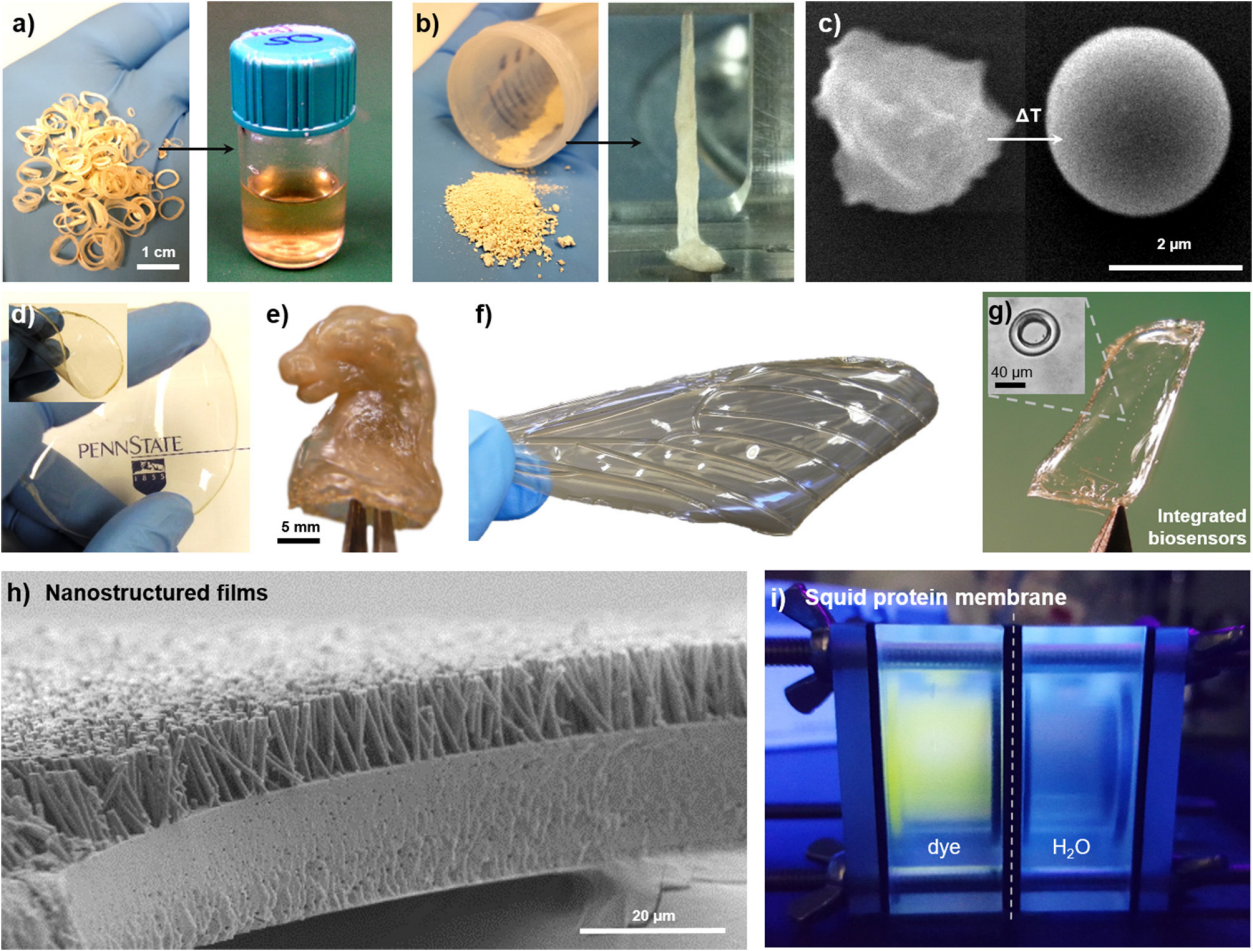
Multifunctional films fabricated from squid-derived proteins. SRT proteins are processed via **(a)** solution-based or **(b)** thermal-based methods into a variety of materials. Reproduced with permission (Pena-Francesch et al., 2014b). Copyright 2014 Wiley. **(c)** Colloids. Reproduced with permission (Pena-Francesch et al., 2014b). Copyright 2014 Wiley. **(d)** Free-standing transparent flexible films. Reproduced with permission (Yilmaz et al., 2017). Copyright 2017 American Chemical Society. **(e)** Complex 3D geometries. Reproduced with permission (Pena-Francesch et al., 2018b). Copyright 2017 American Chemical Society. **(f)** Biomimetic materials. Reproduced with permission (Michaels et al., 2015). Copyright 2015 Springer **(g)** Optical microresonators. Reproduced with permission (Yilmaz et al., 2017). Copyright 2017 American Chemical Society. **(h)** Nanostructured surfaces. Reproduced with permission (Guerette et al., 2013). Copyright 2013 Nature Publishing Group. **(i)** SRT-based membranes.

Due to the reversible and non-covalent nature of the crosslinking mechanism, SRT proteins are available for fabrication and processing methods that are common in the polymer industry. Solution-based processing of SRT, for example, consists in disrupting the hydrogen bonding in the β-sheet structures, and solubilizing the protein for posterior solvent casting (Figure 4a). Acidic/basic aqueous solutions, salts, surfactants, and organic solvents are typically used to accelerate the disruption of β-sheets and increase the protein solubility (solvent residues can be easily washed away from the final product after evaporation) (Pena-Francesch et al., 2018a). On the other hand, thermoplastic processing of SRT proteins is also possible by heating the protein material above its glass transition temperature (Figures 4b,c; Pena-Francesch et al., 2014b). The glass transition temperature of SRT can be tailored to the desired processing conditions by optimization of the nanostructure and use of plasticizers, opening up processing capabilities traditionally restricted to synthetic materials (extrusion, injection, lamination, etc.) (Pena-Francesch et al., 2018a). Using solution- and thermal-based methods, SRT proteins have been processed into numerous complex materials at the nano-, micro-, and macroscale. Transparent and flexible free-standing SRT films (Figure 4d) were fabricated by drop casting (Pena-Francesch et al., 2014b, 2018a; Jung et al., 2016), and used as substrate, membrane, or support material for multiple applications including but not limited to bioadhesive pads (Pena-Francesch et al., 2014a), fully biodegradable sensors (Yilmaz et al., 2016, 2017), and stretchable proton conductors(Pena-Francesch et al., 2018b). SRT proteins were processed into complex 3D geometries (Figure 4e) by combining solution- and thermal-based techniques (e.g., micro-/nanomolding, nanowetting) (Guerette et al., 2013; Pena-Francesch et al., 2014b, 2018a; Yilmaz et al., 2017). The processing versatility of SRT proteins has allowed the design and fabrication of bioinspired devices and materials, such as insect-inspired wings for flapping wing micro air vehicles (FWMAVs) (Figure 4f). Insects have superior flight maneuverability in close quarters than other flying animals mainly due to the material and structural properties of their wings (Ansari et al., 2006). Insect wings are generally composed of a stiff chitin-based venation structure embedded within a protein membrane, which provide mechanical support and flexibility to the wing (Combes, 2010). A protein-based artificial wing inspired in the hawkmoth *Manduca sexta* was fabricated using SRT proteins (Michaels et al., 2015), demonstrating the potential of SRT proteins in replicating natural systems with biological materials while maintaining the physical and chemical properties of the proteins. Arrays of high-quality optical cavities (such as microresonators for photonic devices and biosensors) have been integrated in flexible protein films via soft lithography and protein molding techniques (Figure 4g; Yilmaz et al., 2016, 2017). SRT nanostructured films, such as nanograss, have been fabricated using template-based nanowetting and capillary micromolding, producing high aspect ratio nanofiber arrays (Figure 4h) that replicate textured surfaces found in nature (lotus leaf, gecko's footpad, butterfly wings, etc.) (Guerette et al., 2013; Pena-Francesch et al., 2014b). SRT-based free-standing thin films have been explored as separation membranes. Thin membranes to remove molecular contaminants in water treatment processes have gained recent attention in the research community due to the growing global problem of water pollution (Shannon et al., 2008; Vandezande et al., 2008). A diversity of new materials and fabrication techniques have been investigated to develop efficient membranes, including nanomaterials biopolymers (cellulose, silk, amyloids) (Bolisetty and Mezzenga, 2016; Ling et al., 2016, 2017; Zhang et al., 2016). However, developing thin, mechanically strong membranes with tunable barrier properties and good separation performance remains a challenge. SRT-materials hold promise in this field due to their mechanical strength, flexibility, tunable nanostructure, and self-healing properties (Pena-Francesch et al., 2014b; Sariola et al., 2015). SRT membranes show a good performance under low flux conditions, with 100% rejection of Rhodamine B dye (Figure 4i; Barbu, 2016).

## SRT Based Films in Textile Applications

Since the dawn of civilization, natural fibers (e.g., wool, cotton, sisal, ramie, silk) were used in textiles. However, due to increased demand and cost issues, synthetic fibers made of polyester, nylon, and others replaced natural alternatives. Recently bio-derived or biosynthetically produced fibers received significant interest due to sustainability and environmental reasons. Although the environmental and health regulations in textile industry have been a driving force for the sustainability movement, novel properties discovered in biosynthetic fibers are also increasing the momentum of this initiative. In this respect, SRT proteins hold great promise to provide a broad range of solutions for the textile industry because of its programmable properties, biodegradability, and easy processing, such as self-healing recyclable fabrics, natural sewing free adhesive, smart garments for health monitoring, and new strategies for the reduction of environmental pollution and health impact.

### Abrasion-Resistant Coatings for Microfibers

**Figure 5:**
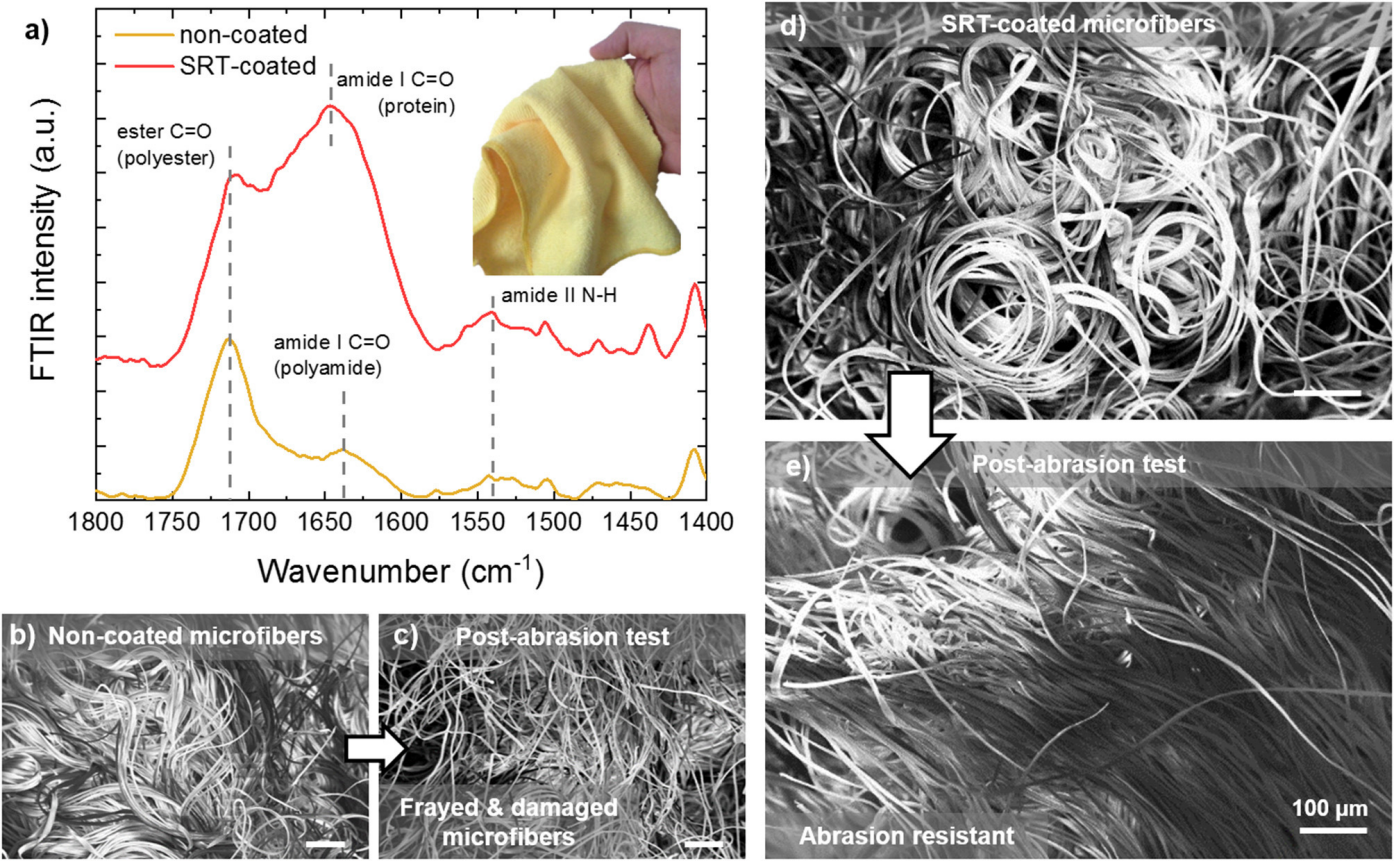
Abrasion-resistant SRT protein coatings for advanced textiles. **(a)** FTIR spectra of SRT-coated and non-coated microfiber cloth. **(b)** Pre-abrasion non-coated microfibers are bundled, but they are **(c)** damaged and frayed after the abrasion test. **(d)** Pre-abrasion SRT-coated microfibers are bundled as well, but **(e)** after the abrasion test the microfibers are aligned and not damaged.

Microplastics (small plastic particles <5 mm in size) are environmental pollutants that are found in freshwater (Dris et al., 2015; Eerkes-Medrano et al., 2015), marine (Cole et al., 2011; Galloway and Lewis, 2016; Gago et al., 2018), and terrestrial environments (Rillig, 2012). Once released in the environment, microplastics are ingested by local organisms (Watts et al., 2015, 2016; Sussarellu et al., 2016), resulting in the intake of toxic chemicals that have a negative impact on marine life and can enter the human water and food supply (Mathalon and Hill, 2014; Yang et al., 2015; Koelmans et al., 2016; Wardrop et al., 2016). Microplastics are originated from primary sources such as microbeads in cosmetics or secondary sources such as the breakdown of larger plastic debris. Synthetic microfibers are generated from washing cycles of common garments (e.g., sub-millimeter size of polyester, acrylic, and nylon fibers), and are then discharged to sewers or surface waters (Hartline et al., 2016). Wastewater treatment plants cannot completely filter microfibers due to their small size, and their efficient removal from effluents represents a major technological challenge in the protection of the environment (Murphy et al., 2016). Recent research has focused not only in improving filtration efficiency and removal of microplastic pollutants from the environment, but also in preventing their generation and release in the first place. With this problem in mind, SRT protein fibers and films have been explored as a potential solution for minimizing microplastic pollution. In Figure 5, a microfiber cloth (87% polyester, 13% polyamide) is coated with SRT protein film, and the microfiber resistance to mechanical damage (i.e., abrasion) is tested. The protein coating was examined by Fourier transform infrared spectroscopy (FTIR), revealing a successful homogeneous coating as shown in Figure 5a. The measured spectrum is consistent with those of previously reported polyester/polyamide fibers (Marjo et al., 2017). Electron microscopy showed the microstructure of the coated and non-coated microfiber cloths are significantly different in their morphologies after wear and tear test. Non-coated cloths showed bundles of microfibers homogeneously distributed over the cloth surface (Figure 5b). However, after the abrasion test, the bundles are frayed and damaged, and individual microfibers are broken and released (Figure 5c). SRT-coated microfibers are also arranged in bundles (Figure 5d), similarly to non-coated fibers. However, SRT-coated fibers do not break after the abrasion test and are not detached from the cloth (Figure 5e). Interestingly, the microfibers align in the direction of the force applied during the abrasion test. These findings suggest that SRT coatings provide mechanical stability to microfibers, could potentially prevent release of microfibers to the environment after mechanical abrasion.

### Self-Healing SRT Films

**Figure 6:**
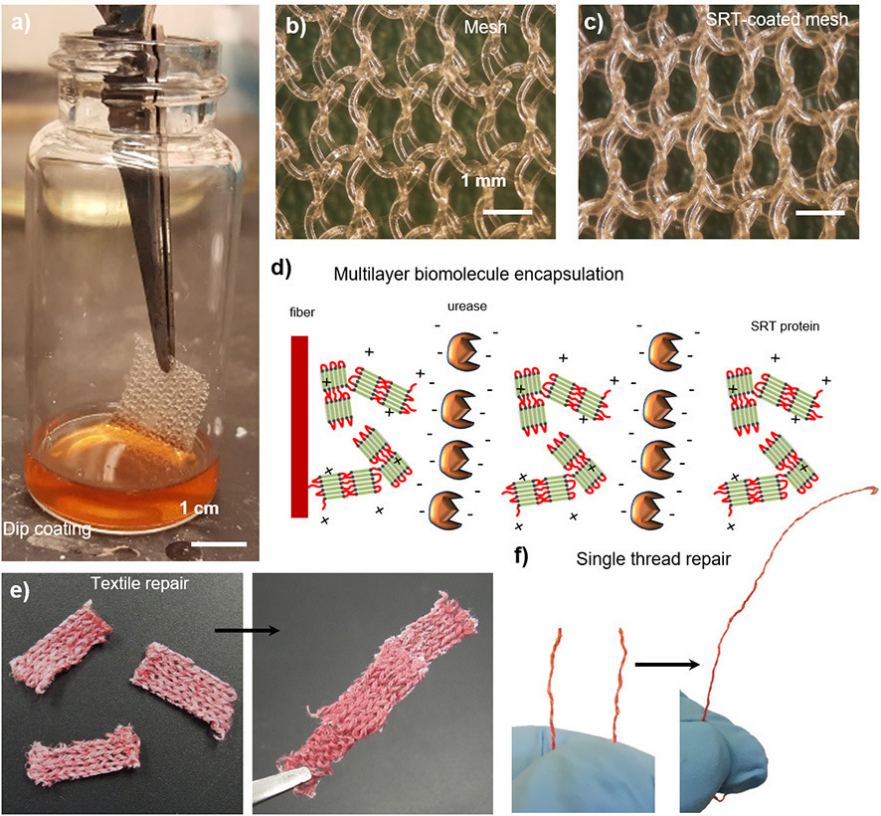
SRT protein films for smart repairable textiles. **(a)** Natural and synthetic fabrics are coated with SRT protein. **(b)** Pre-coating and **(c)** post-coating images show a homogeneous coating around individual polypropylene fibers. Reproduced with permission (Leberfinger et al., 2018). **(d)** Multilayer biomolecule encapsulation (enzymes), providing built-in detection and protection against hazardous agents. Self-healing and repairable **(e)** fabrics and **(f)** single fiber maintain the activity of encapsulated biomolecules. Reproduced with permission (Gaddes et al., 2016). Copyright 2016 American Chemical Society.

Smart textiles that are capable of autonomous self-healing represent an increasingly important class of advanced materials for substrates prone to damage, such as biomedical implants or garments tailored for protection against chemical and biological warfare agents (Lee et al., 2003; Singh et al., 2004). Because of their biocompatibility and self-healing properties, SRT films are good candidates for developing such advanced textiles. A broad variety of textiles can be easily coated with SRT proteins by dip coating, including woven, non-woven, and single fibers (Figure 6a). SRT proteins homogeneously coat the fibers with controllable thickness by adjusting the coating process (i.e., solvent, protein concentration, viscosity, and drying) as shown in Figures 6b,c. Moreover, SRT coatings allow for multilayer biomolecule encapsulation such as enzymes, enabling smart textile applications in biosensing, drug delivery, and chemical/biological warfare protection by enzymatic neutralization. Urease enzymes were used as model enzyme in these studies and were successfully encapsulated in SRT-coated textiles using layer-by-layer deposition, providing built-in multifunctionality (Figure 6d; Gaddes et al., 2016). Stable enzyme-doped SRT films were reproducibly deposited on textile substrates to form a composite that resists dry cracking, repairs macroscopic textile tears in the presence of water, and maintains urease enzyme activity (Figure 6e). Multilayer enzyme/SRT self-healing coatings can be applied not only to textiles but also to single fibers and threads (Figure 6f), which can be later woven into multifunctional smart garments with multiple advanced fibers.

## SRT Proteins for Soft Photonics

**Figure 7:**
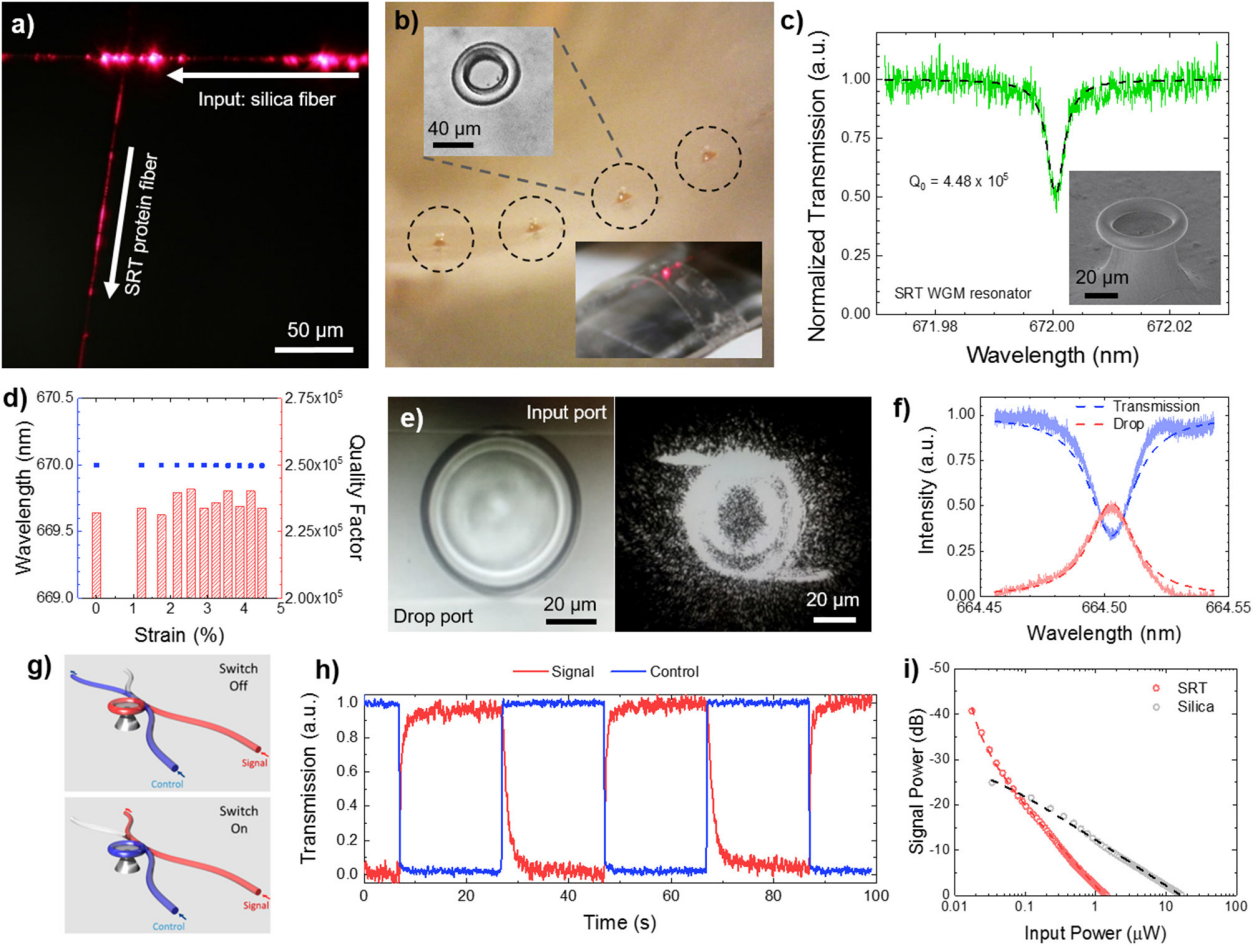
Protein-based soft photonics. **(a)** All-protein optical waveguides, **(b)** protein whispering gallery mode microresonator arrays on flexible substrates, **(c,d)** high quality factor for protein resonators, with stable performance under bending deformation, **(e,f)** SRT-based add-drop filter, **(g–i)** SRT-based optical on/off switch. Reproduced with permission (Yilmaz et al., 2017). Copyright 2017 American Chemical Society.

Multifunctionality in nanostructured SRT films is not limited to passive surfaces, but also includes the incorporation of active sensing capabilities. Photonic devices are typically manufactured with conventional hard materials (silica, silicon, silicon nitride, glass, and quartz) by using standard lithography techniques (Armani et al., 2003). However, these materials are not suitable for applications that require soft, flexible, biocompatible, and biodegradable photonic devices and structures such as *in vivo* biosensing and biodetection. Optical wave-guiding capabilities of flexible protein-based fibers were demonstrated by coupling light from silica fiber taper to SRT fibers (Figure 7a), opening up new functionalities of SRT as soft photonics platforms. All-SRT photonic platforms have been fabricated by integrating whispering-gallery-mode (WGM) microresonators in flexible protein films (Figure 7b). SRT proteins proved an excellent soft material for WGM biophotonic platforms, with quality factors as high as 10^5^, and two orders of magnitude larger thermo-optic coefficient than silica (Figure 7c; Yilmaz et al., 2017). Furthermore, the resonance wavelength and quality factor of SRT WGM flexible resonators remained unaffected when the substrate film was bent, making SRT-based microresonators an attractive platform for biologically integrated sensing (Figure 7d). To exploit these promising optical properties, we designed and fabricated SRT-based soft photonic devices. We fabricated add-drop filters (optical communication architectures) by coupling two separate fiber-taper waveguides to protein WGM resonators (Figure 7e; Yilmaz et al., 2017). Non-resonant light passes through the input waveguide to the transmission port. The filter performed with an efficiency of 51% (Figure 7f), which can be increased by improving the waveguide-resonator coupling and decreasing scattering losses. Furthermore, photonic on/off switches were fabricated from SRT protein (Figure 7g; Yilmaz et al., 2017). The transmission of a signal field through a waveguide-coupled microresonator was switched between on and off states by a control field via the thermal response of SRT proteins (hence, exploiting the strong thermo-optic coefficient of the proteinaceous material) (Figure 7h). All-SRT switches achieved an isolation of 41dB at a control field power of 1.44 μW (circulating power 0.129 mW (Figure 7i). Compared to an all-silica switch (25 dB isolation, 16.43 μW control power, 219.76 mW circulating power), SRT-based switches are 14x more energy efficient than their “hard” equivalents (due to their strong thermo-optic coefficient and negative thermal expansion). Therefore, protein-based soft, flexible, biodegradable photonic devices are attractive for low power consumption applications such as biosensing.

## Tandem Repeat Protein Based 2D Layered Nanocomposites

**Figure 8:**
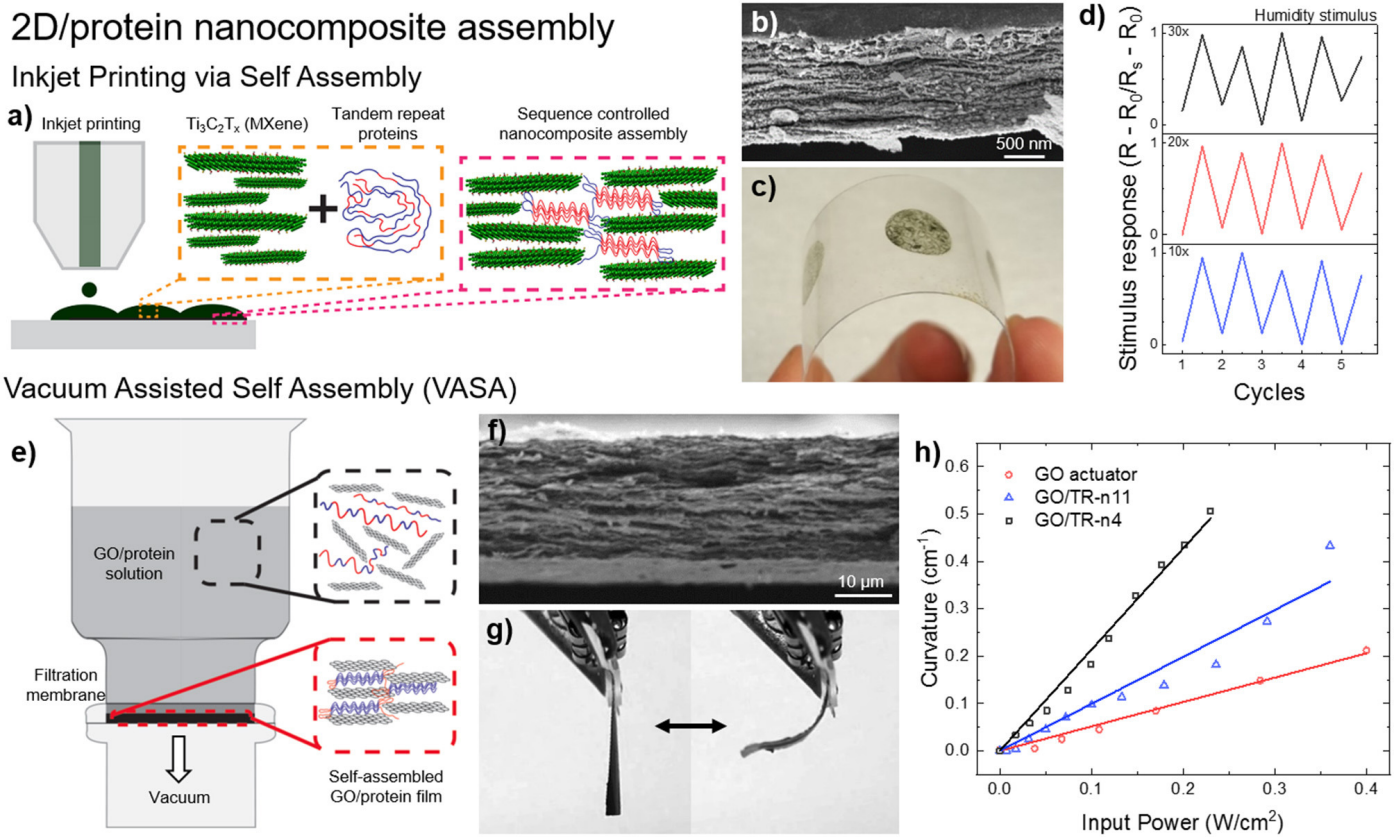
Protein-based 2d-layered nanocomposites. **(a)** Inkjet printing self-assembly of **(b)** nanostructured, **(c)** flexible MXene-SRT protein electrodes and their **(d)** electrical response to environmental humidity. Reproduced with permission (Vural et al., 2018). Copyright 2018 Wiley. **(e)** Vacuum assisted self-assembly (VASA) of **(f)** graphene oxide (GO) and SRT proteins for the fabrication of **(g)** programmable bimorph thermal actuators with **(h)** high efficiency. Reproduced with permission (Vural et al., 2017). Copyright 2017 Elsevier.

Tandem repeat proteins play an important role for creating composite structures in nature such as nacre. Similarly, these proteins are recently used for dynamic assembly of 2d layered structures as shown in Figure 8 (Demirel et al., 2018). For example, nanocomposite films of SRT proteins with 2d-layered MXene structures were demonstrated in stimuli responsive flexible electronic films via inkjet printing self-assembly (Figure 8a; Vural et al., 2018). MXenes are conductive materials that have the general formula of M_n+1_X_n_T_n_ (M is an early transition metal, X is carbon or nitrogen, T_x_ stands for surface functional groups [-F, -O, -OH] and *n* = 1–3). Their high electrical conductivity and electromagnetic interference shielding efficiency (EMI SE) can be harnessed as printed electrodes. Tandem repeat proteins inspired by SRT played a significant role as promising binders between MXene 2d layers via hydrogen bonding with surface termination groups of Mxenes (-F, -O, -OH) as well as a stabilizer for printable conductive inks. Inkjet printed electrodes of SRT-MXene exhibits superior electrical conductivity values as high as 1080 S/cm on flexible polyethylene terephthalate (PET) substrate, which is meaningfully higher than other two-dimensional materials such as graphene (250 S/cm) and reduced graphene oxide (340 S/cm). These electrodes demonstrated stimuli responsive (e.g., humidity) metal-insulator transitions through percolation of conductive layer. The electrodes of Ti_3_C_2_T_x_-SRT exhibit on/off respond to humidity change which is desirable for humidity sensors. Moreover, electromagnetic interference (EMI) shielding ability of printed electrodes was also demonstrated. Printed electrodes with protein concentration of 0.95 mg/ml inks show EMI SE values as high as 50 dB for an electrode thickness of 1.35 μm between 8 and 12 GHz at ambient humidity (60% RH). As another example, vacuum assisted self-assembly (VASA) of highly ordered 2D composites based on graphene oxide (GO) attracted interest in applications that require high mechanical strength as well as increased thermal conductivity (Figure 8b; Vural et al., 2017). A wide spectrum of tandem repeat proteins has been utilized to fabricate GO-protein composites such as elastin-like protein, nacre-like gelatin, silk fibroin and SRT. Additionally, by manipulating the interlayer distance of GO 2d layered composites, bimorph thermal actuators have been fabricated combining the high thermal conductivity of GO (300 W/mK) and the high thermal expansion coefficient of SRT proteins (−95 × 10^−6^ K^−1^). GO sheets are responsible for homogenous heat dispersion, whereas the tandem repeat proteins trigger thermal expansion. Compared to regular GO actuators, protein-GO 2d layered composites showed 1800x higher performance enhancement of thermal actuation. In summary, assembly and control of 2d-layered/protein composites could find applications in next-generation, programmable, flexible, optically and electrically superior, energy efficient and mechanically strong materials and devices such as 2d heterostructures for topological electronics, mottronics, photonics, and spintronics including but not limited to: engineering spectrum of physical properties such as direct bandgap, strong spin-orbit coupling, optical non-linearities, and photoconductance, extraordinary electronic and optical properties such as thin-film photodetectors, logic memory devices, transistors, photovoltaics, and supercapacitors.

## Author Contributions

All authors listed have made a substantial, direct and intellectual contribution to the work, and approved it for publication.

### Conflict of Interest Statement

The authors declare that authors have issued and pending patents.
